# Comprehensive Analysis of Elastic–Plastic Behavior in Hybrid Metal Matrix Composites with Varied Reinforcement Geometry

**DOI:** 10.3390/ma18122763

**Published:** 2025-06-12

**Authors:** Grzegorz Mieczkowski, Dariusz Szpica, Andrzej Borawski

**Affiliations:** Faculty of Mechanical Engineering, Bialystok University of Technology, 45C Wiejska Str., 15-351 Bialystok, Poland; g.mieczkowski@pb.edu.pl (G.M.); a.borawski@pb.edu.pl (A.B.)

**Keywords:** Hybrid Metal Matrix Composites (HMMCs), effective mechanical properties, mechanical property prediction, elastic–plastic behavior, finite element method (FEM), discontinuous reinforcement

## Abstract

This study presents a comprehensive analytical–numerical approach to determining the elastic–plastic properties of Hybrid Metal Matrix Composites (HMMCs), contrasting with prior research that primarily emphasizes elasticity. Using the finite element method (FEM) and elasticity and plasticity theory, we determined key parameters, including Young’s modulus, Poisson’s ratio, yield strength, and ultimate tensile strength. The method, which also accounts for strain-hardening behavior via the Hollomon model, enables precise simulation of HMMC with randomly distributed reinforcement particles of varying shapes and sizes, offering a realistic representation of the composite microstructure. Verification against the literature confirms the accuracy of the approach in reflecting both elastic and plastic behavior, providing essential insights into the material’s full mechanical response, particularly yield strength and strain-hardening properties, aspects rarely explored in depth in existing studies on HMMCs.

## 1. Introduction

In recent years, there has been a notable and continuous increase in the utilization of metal matrix composites across various industrial sectors, including defence [1], automotive [2,3], and aerospace [4] industries. These composites demonstrate superior mechanical and performance properties compared to homogeneous materials, such as ceramics, metals, and plastics. This superiority is attributed to the combination of the optimal properties of the matrix (e.g., toughness and fracture toughness) and reinforcement (e.g., high strength, elastic modulus, and wear resistance) [5,6].

The mechanical properties of individual components of a composite are crucial determinants of its strength and functionality. However, an equally significant factor is the structure of the composite, which is dependent on manufacturing technology [7,8,9]. The morphology, dimensions, volume proportion, and distribution of the reinforcement particles influence parameters such as the Young’s modulus [10,11], yield strength, tensile strength [12,13,14], and fracture toughness [15,16]. Moreover, insufficient interparticle spacing or acute corners of the reinforcement particles can generate localized stress concentrations, potentially leading to crack initiation [17,18].

Experimental studies are the most reliable method for determining the effective mechanical properties of composites. Interesting results on composites with a single reinforcement (SiC, Al_2_O_3_) have been presented in papers [19,20,21,22]. Hybrid composites containing more than one type of reinforcement (e.g., fibers, short fibers, particles, and whiskers) have been gaining importance since the 1970s. Studies on their properties can be found in works [23] (different particle shapes) and [24,25] (different materials and particle shapes).

Even small changes in the geometric parameters of a composite can significantly affect its mechanical properties. The use of experimental methods to predict these properties involves considerable effort and costs. Therefore, it is necessary to develop alternative prediction methods.

Analytical or analytical–numerical models can be used as alternatives to experimental studies. Owing to their simplicity, analytical models only allow the determination of the basic or limiting properties of composites. In previous studies [26,27,28], formulae were presented to determine the limiting values of the elastic modulus and Poisson’s ratio, taking into account the variation in the volume proportion of reinforcement. These models assumed the presence of one type of reinforcement with a constant particle size.

With the development of numerical methods, the finite element method (FEM) has become increasingly popular. FEM simulations can be used for strength tests [29,30,31,32,33,34,35], friction [36,37], fluid flow [38,39,40], heat [41], and piezoelectricity [42,43,44]. The FEM is also useful for determining the effective properties of composite materials. Compared with experimental studies, FEM simulations are cost-effective and less time consuming. The advantage of the FEM over analytical modeling is its ability to accurately represent the shape, size, and distribution of reinforcement particles without the need for simplification. Interesting studies on the estimation of the effective properties of single-reinforcement composites (using the FEM) can be found in papers [45,46,47,48,49]. The authors of papers [45,46,47] modeled composites with discontinuous reinforcement as two-dimensional (2D) problems. In principle, this approach allows only composites reinforced with spherical particles to be modeled. Spatial models are necessary for composites with different scattered fraction shape, spatial models are necessary [48,49]. Three-dimensional models can also be used for composite structures in which the reinforcement particles are spherical [50].

The modeling of hybrid composites with complex structures (varying shapes and sizes of reinforcement particles) has rarely been reported in the literature. Study [51] developed an FEM-based RVE to analyze the thermal properties of hybrid epoxy/E-glass/H-glass hollow sphere composites, focusing on thermal conductivity and CTE. Study [52] used homogenization and the Halpin–Tsai model to predict the elastic moduli of nano-hybrid epoxy composites reinforced with CNTs and nano-clay. Neither of these works addresses any aspect of plastic deformation or hardening.

The present study introduces an analytical–numerical method for determining the properties of such structures. This method allows the estimation of parameters such as density, Young’s modulus, Poisson’s ratio, yield strength, and tensile strength, as well as parameters describing the strain-hardening behavior of the material according to Hollomon’s model. By employing this method, hybrid composites with different forms of reinforcement can be modeled with any volume fraction, as reinforcement particles are permitted to vary in shape (spheres, cylinders, or ellipsoids), size, and distribution. The proposed method is distinguished by the integration of advanced representative volume element (RVE) generation with a comprehensive elastic–plastic analysis.

In contrast to many previous studies that focused solely on either the modeling of elastic properties or on microstructure generation using tools such as Mérope [53] or the Sequential Addition and Migration methods [54], the present method enables the simultaneous determination of both elastic and plastic parameters. Notably, the effective Young’s modulus, yield strength, and tensile strength obtained from numerical simulations exhibit excellent agreement with experimental results, with discrepancies of less than 3%, thus confirming the accuracy and reliability of the approach.

Simulation results further indicate that particle geometry plays a key role in determining the stress distribution, as the presence of sharp particles (such as cylinders and ellipsoids) leads to higher stress concentrations and earlier onset of plasticity.

In this study, a detailed analysis of the effect of the geometric parameters of the reinforcement particles on the properties of hybrid composites was also conducted. Composites with an aluminium matrix (HAMCs) and triple reinforcement were analyzed by varying the shapes and sizes of the reinforcement particles, with the results discussed in Section 3. Future work is planned to extend the model to include failure strain analysis, which will allow for a complete characterization of the composite’s damage behavior by incorporating appropriate damage criteria for the matrix.

## 2. Method for Determining the Effective Mechanical Properties of Hybrid Composites

### 2.1. Method Description

Numerical methods are attractive alternatives to experimental and analytical methods for determining the effective mechanical properties of composites. Averaged parameters, such as density, elastic modulus, Poisson’s ratio, and elastic–plastic properties (yield strength, tensile strength, and parameters describing the stress–strain curve), were determined from the properties of the homogeneous material phases and their geometry (volume fraction, shape, and size of reinforcement particles).

In this study, the finite element method (FEM) was used. The process of determining the averaged parameters of the hybrid composites involved three steps. In the first stage, a three-dimensional model of the composite microstructure was created by considering the shape, size, and volume fraction of the reinforcement particles. In the second stage, the model was discretized, boundary conditions (fixing and loading) were imposed, and simulations were conducted to determine the stress and strain states. In the final and third stages, the desired mechanical properties were calculated from the simulation results using the constitutive equations of elasticity and plasticity theory [55], material hardening principles [56], and strength hypotheses [57]. A detailed description of these steps is provided below.

#### 2.1.1. Modeling the Microstructure of the Composites

**Figure 1:**
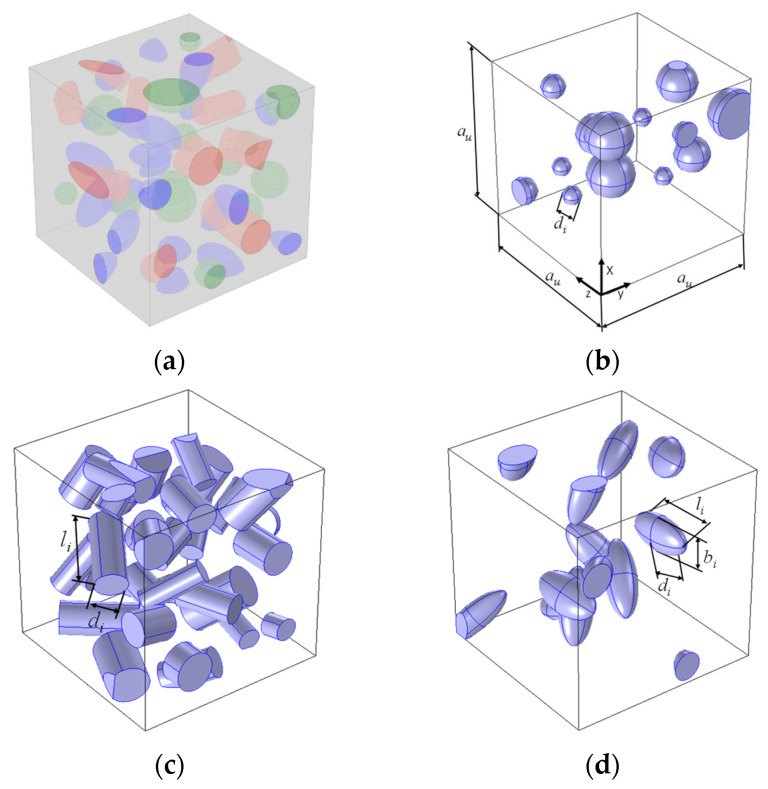
Representative volume elements: (**a**) hybrid composite with three types of reinforcement, (**b**) composite with spherical reinforcement (volume fraction of reinforcement 10%), (**c**) composite with cylindrical reinforcement (volume fraction of reinforcement 20%), and (**d**) composite with ellipsoid reinforcement (volume fraction of reinforcement 10%).

The geometrical models assume that the representative volume element (RVE) (Figure 1a) is in the form of a cube with a side equal to *a_u_*. The composite contained several different reinforcement fractions. Reinforcement particles can take the form of spheres (Figure 1b), cylinders (Figure 1c), or ellipsoids (Figure 1d). The matrix, shown in grey in Figure 1a, fills the cube space that is not occupied by the reinforcement. The geometrical dimensions of solids corresponding to the reinforcement particles can vary randomly within a certain range.

- sphere: $d_{i}\in [d_{\min},d_{\max}]$;
- cylinder: $d_{i}\in [d_{\min},d_{\max}]$, $l_{i}\in [l_{\min},l_{\max}]$;
- ellipsoid: $d_{i}\in [d_{\min},d_{\max}]$, $b_{i}\in [b_{\min},b_{\max}]$, $l_{i}\in [l_{\min},l_{\max}]$.

**Figure 2:**
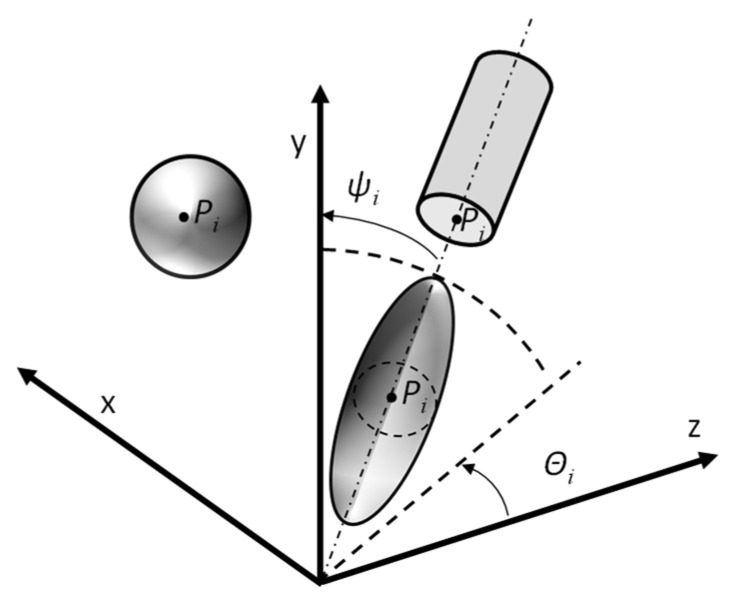
Modeled single reinforcements as solids in 3D space.

The location of the individual bodies in the model (Figure 2) was determined randomly, which means that the position of point *P_i_* (*x_i_*, *y_i_*, *z_i_*) ($x_{i},y_{i},z_{i}\in [0,a_{u}]$), representing the centre of the sphere, ellipsoid, or circle of the lower base of the cylinder, was determined randomly. For the cylinder- and ellipsoid-shaped reinforcement, two Euler angles were additionally randomly assigned: *θ_i_* and *ψ_i_* (*θ_i_*, *ψ_i_* ∈[0,2π]). An important assumption is that the solids that represent the reinforcement particles cannot intersect. To ensure this, the distance between the axes of the newly added elements and existing ones was calculated when generating new solids. By “axis of a solid” was meant:

- for a sphere: a segment of length *d_i_* whose centre coincides with the centre of the sphere (sphere diameter);
- for a cylinder: a segment of length *l_i_* connecting the centres of the circles of the lower and upper bases (the height of the cylinder)
- for an ellipsoid: a segment of length *l_i_*, whose centre coincides with the centre of the ellipsoid (the longest axis of the ellipsoid).

The distance between the axes cannot be less than 1.1·*d_max_*. The distance between the solid axes was calculated as previously described [58,59].

When the solid exceeded the set boundaries of the composite fragment (cube of side *a_u_*), the protruding part was cut off and the volume contribution of the reinforcement was revised.

**Figure 3:**
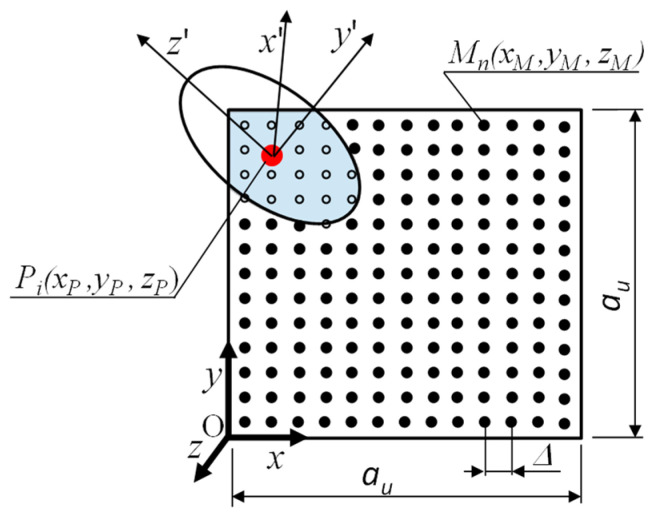
Sampling in numerical integration method, •—points outside the area of the reinforcement particle, °—points lying inside the reinforcement particle.

A numerical integration method based on a regular mesh [60] was used to estimate the volume that remained after the cut-off (grey in Figure 3). The representative volume element was discretized into N points, whose coordinates in the global reference system (O, *x*, *y*, *z*) varied with a constant step Δ. The volume of solid remaining in the RVE was determined using the following formula:

(1) $$V_{a}=\frac{N_{i}}{N}a_{u}^{3},$$

where *V_a_* is the volume of the solid remaining in the RVE, *N* is the total number of points used in the sampling, and *N_i_* is the number of points inside the reinforcement particle. Point *M_n_* was assumed to lie inside the reinforcement particle when the following conditions were met:

- for the spherical reinforcements

(2) $$\left|\vec{P_{i}M_{n}}\right|\leq d_{i}/2,$$

- for the cylindrical reinforcements

(3) $$\left\{\begin{array}{l} {x^{\prime }}_{M}^{2}+{y^{\prime }}_{M}^{2}\leq {\left(\frac{d_{i}}{2}\right)}^{2}\\ 0\leq z_{M}^{\prime }\leq l_{i} \end{array}\right.,$$

- for ellipsoid-shaped reinforcement

(4) $$\frac{{x^{\prime }}_{M}^{2}}{{\left(d_{i}/2\right)}^{2}}+\frac{{y^{\prime }}_{M}^{2}}{{\left(b_{i}/2\right)}^{2}}+\frac{{z^{\prime }}_{M}^{2}}{{\left(l_{i}/2\right)}^{2}}\leq 1,$$

where *d_i_*, *b_i_*, and *l_i_* are the characteristic dimensions of the solids representing the reinforcement material and $x_{M}^{\prime },\;y_{M}^{\prime },\;z_{M}^{\prime }$ represents the coordinates of the sampling points in the local reference system, the origin of which is located at point *P_i_* (Figure 3). The transformation of the coordinates of the *M_n_* points from the global system (O, x, y, z) to the local system ($P_{i},x^{\prime },\;y^{\prime },z^{\prime }$) was performed using the following formula:

(5) $$\left[\begin{array}{c} {x^{\prime }}_{M}\\ {y^{\prime }}_{M}\\ {z^{\prime }}_{M} \end{array}\right]=A_{1}^{3\times 3}\times A_{2}^{3\times 3}\times \left[\begin{array}{c} x_{M}-x_{P}\\ y_{M}-y_{P}\\ z_{M}-z_{P} \end{array}\right],$$

where *x_P_*, *y_P_*, and *z_P_* are the coordinates of point *P_i_* (the base point of the solids corresponding to the reinforcement material) in the global reference system and $A_{1}^{3\times 3}$ and $A_{2}^{3\times 3}$ are the matrix of rotation for the given Euler angles (Figure 2). The matrixes were defined using the following formulae:

(6) $$A_{1}^{3\times 3}=\left[\begin{array}{ccc} 1 & 0 & 0\\ 0 & \cos(\psi ) & -\sin(\psi )\\ 0 & \sin(\psi ) & \cos(\psi ) \end{array}\right],$$

(7) $$A_{2}^{3\times 3}=\left[\begin{array}{ccc} \cos(\theta ) & 0 & \sin(\theta )\\ 0 & 1 & 0\\ -\sin(\theta ) & 0 & \cos(\theta ) \end{array}\right],$$

**Figure 4:**
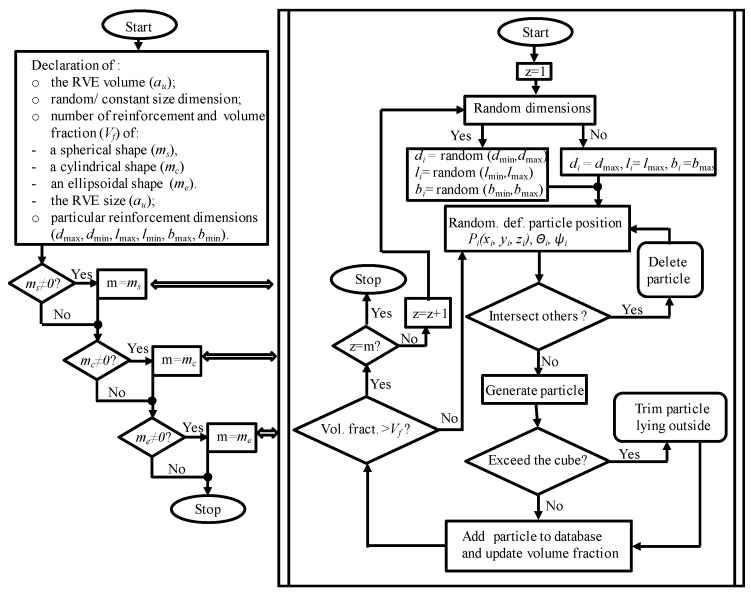
Algorithm of the program for generating the RVA spatial model.

In all developed RVE models, the reinforcement particles were assumed to be connected to the matrix without any intermediate layers. To obtain the assumed distribution of the reinforcement components, it was necessary to develop a proprietary program written in Java for automatic generation of the composite microstructure. The algorithm of the program is illustrated in Figure 4.

The assumption of random particle placement was made because detailed imaging data (e.g., from SEM) for the particular HMMC variants were unavailable. We recognize that a direct comparison with actual micrographs could further validate the RVE model. The random-placement algorithm also assumes that each inclusion is fully surrounded by matrix material. In practice, this condition can break down once the volume fraction of hardening particles approaches or exceeds the percolation threshold. At higher concentrations, clusters of particles may form—with no or minimal intervening metal—leading to strong local interactions. These interactions produce elevated stress concentrations and can initiate microcracks at particle–particle contacts. Because our current model does not explicitly resolve such high-density clusters or account for microcrack nucleation, its predictions are most reliable for inclusion fractions below the percolation limit.

#### 2.1.2. FEM Modeling

One key advantage of FEM over many analytical models is its ability to accurately represent the shape, size, and distribution of reinforcement particles without the need for simplification. Although methods based on the Fast Fourier Transform (FFT) have emerged as an efficient alternative—particularly enabling simulations of larger-volume elements with periodic microstructures [61]—such approaches are typically better suited for materials with highly regular and periodic microstructures. In contrast, the present study focuses on hybrid composites with complex, random reinforcement geometries, where FEM is better suited to capture local stress concentrations and the nonlinear elastic–plastic behavior of the material.

The 3D models prepared in the first stage were used in the numerical FEM calculations, which were performed using the COMSOL Multiphysics software 5.6. Solid models of the composite were described using quadratic tetrahedral finite elements, a choice made by the software based on the physical phenomena under investigation, ensuring an optimal balance between accuracy and computational efficiency. These elements were applied with increased mesh density at the interfaces between the matrix and reinforcement, where stress concentrations typically occur and accurate modeling is critical. Mesh quality was verified using COMSOL’s built-in tools, and a convergence study with coarse, medium, and fine meshes showed that the medium mesh yielded results identical to the fine mesh.

**Figure 5:**
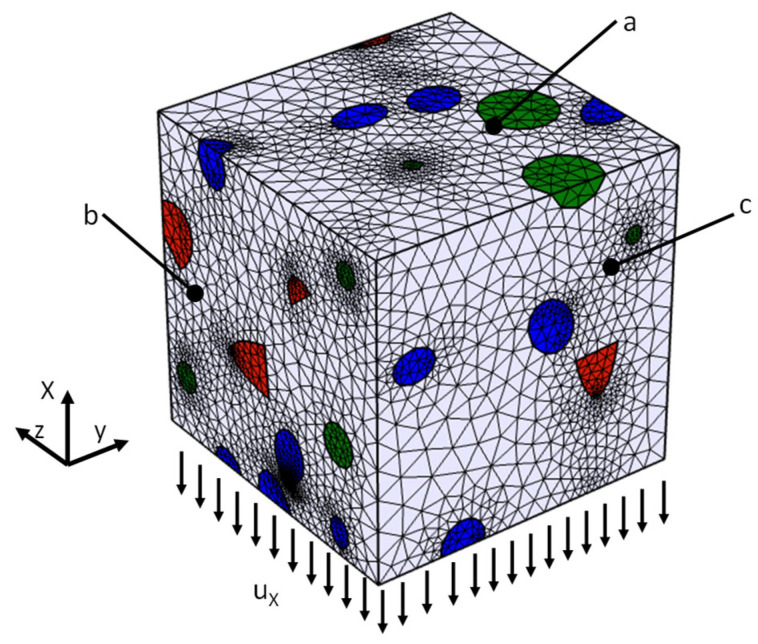
Boundary conditions with finite element division mesh and applied boundary conditions.

Moreover, the software automatically refines the mesh in critical regions, such as geometric discontinuities, which enhances the precision of the results. A series of preliminary studies with varying element sizes were conducted, and it was observed that further refinement beyond a certain mesh size did not significantly affect the results. Consequently, smaller elements were not employed because they would have unnecessarily prolonged the computational time without yielding additional accuracy. This approach provided an efficient and precise solution to the problem at hand. The finite element mesh and the applied boundary conditions are shown in Figure 5.

Symmetry boundary conditions were applied to walls a, b, and c, while the wall opposite of wall a was subjected to a controlled displacement (u_x_) to simulate uniform deformation. Although fully periodic boundary conditions can further reduce boundary effects—as demonstrated in works [62,63,64]—the present approach was designed to retain the inherent randomness of the hybrid composite microstructure. This quasi-periodic strategy, based on symmetry, simplifies the algorithm and permits the generation of multiple statistically representative realizations. It is noted that periodic generators, such as those described in [53,54,65], are readily available for various inclusion geometries; however, they are typically optimized for less complex systems. Full periodicity would require imposing a perfectly regular, periodic arrangement of particles, which does not reflect the actual random microstructure of HMMCs. Using symmetry conditions on the RVE faces, together with analysis over several independent realizations, ensures statistical representativeness of the microstructure, as confirmed by comparison with experimental data (error < 3%).

Two types of analyses were performed: static and quasi-static. In static analyses, a constant displacement u_x_ =const. was applied, and the results were used to determine the basic effective mechanical–physical properties of the composite, such as Young’s modulus, Poisson’s ratio, and density.

In the quasi-static simulations, the displacement u_x_ was varied in steps. For each value of applied displacement, the stresses and strains in the RVE were calculated. The strains and stresses obtained from the earlier step were then summed with the calculated values. This approach enabled the development of stress–strain curves, which were necessary to determine the averaged elastic–plastic properties of the hybrid composite (yield stress, ultimate tensile strength, and material constants describing material hardening).

Each fraction of the hybrid composite, both the matrix and reinforcement, was assigned unique elastic–plastic properties. Two material models were used in the simulations.

I. The linear elastic material model for all composite components (static analysis);

II. The linear elastic model for the materials used as reinforcements and the elastoplastic model (obeying the von Mises yield criterion [57] for the material from which the matrix is made (quasi-static analysis).

Young moduli (*E_i_*), Poisson’s coefficients (*ν_i_*), and density (*ρ_i_*) were defined for all materials forming the composite. For the matrix, the yield stress (*σ_ys,_*_0.2_), ultimate tensile strength (*σ_u_*), and parameters describing the strain-hardening in the material (Hollomon model) were defined as the strain hardening exponent (*n^H^*) and strength coefficient (*K^H^*).

In the present study, it is assumed that the reinforcements are perfectly bonded to the matrix. Accordingly, continuity of displacement and traction is enforced across the reinforcement–matrix interface, ensuring that no debonding, slip, or separation occurs. This idealized bonding condition implies that the reinforcement and the matrix deform as a single continuous material, which facilitates efficient load transfer between the phases. The perfect bonding assumption is consistent with previous studies where interfacial effects are neglected to focus on the overall mechanical response of the composite. This modeling approach has been applied uniformly throughout the analysis and is further validated by the close agreement between the simulation results and experimental data.

In terms of computational effort, the static simulations were relatively short, typically completing in around 2 min on a standard PC for a homogeneous material model. However, static analyses of composite models (with heterogeneous microstructures) took roughly 4–8 times longer than their homogeneous counterparts. Quasi-static simulations were significantly more time-consuming: a quasi-static analysis for a homogeneous model required on the order of 30 min of runtime under the same conditions. Incorporating composite heterogeneity further increased the cost, with quasi-static runs for composite models taking approximately 15–35 times longer than the homogeneous cases, depending on the complexity of the model.

As mentioned earlier, the objective of the numerical calculations was to obtain a quantitative description of the stress–strain distribution in an RVE.

#### 2.1.3. Determination of Elastic and Elastic–Plastic Properties of Hybrid Composites

The mechanical and physical properties of the composites were determined using the basic equations of elastic and plasticity theories. Although these equations are usually applied to homogeneous materials, they can also be used for composites, provided that the averaged stress and strain parameters are used [66]. Assuming that the *V*_RVE_ and *dV*_RVE_ represent the total and elemental volumes of the RVE, respectively, the averaged stress and strain can be expressed as follows:

(8) $${\bar{\sigma }}_{ij}=\frac{1}{V_{\mathrm{RVE}}}\int_{V_{\mathrm{RVE}}}\sigma_{ij}\;dV_{\mathrm{RVE}},\;{\bar{\varepsilon }}_{ij}=\frac{1}{V_{\mathrm{RVE}}}\int_{V_{\mathrm{RVE}}}\varepsilon_{ij}\;dV_{\mathrm{RVE}},$$

where *σ_ij_* and *ε_ij_* are the local stress and strain in the RVE, respectively.

The averaged values of these quantities were determined using the finite element method.

To determine the basic mechanical–physical properties of the hybrid composites, the relationships (9)–(11) and data obtained from the static FEM analysis were used:

- effective Young’s modulus (Hook’s law)

(9) $$\bar{E}=\frac{{\bar{\sigma }}_{x}}{{\bar{\varepsilon }}_{x}},$$

- effective Poisson’s ratio (change in the material volume)

(10) $$\bar{\nu }=-\frac{dV-{\bar{\varepsilon }}_{x}}{2{\bar{\varepsilon }}_{x}},dV={\bar{\varepsilon }}_{x}+{\bar{\varepsilon }}_{y}+{\bar{\varepsilon }}_{z},$$

- effective density

(11) $$\bar{\rho }=\frac{1}{V_{\mathrm{RVE}}}\int_{V_{\mathrm{RVE}}}\rho_{i}dV_{\mathrm{RVE}},$$

where *ρ_i_* is the density of the matrix material and individual reinforcement components.

In addition to the basic properties, the effective elastic–plastic properties of the composites were determined. Stress–strain curves ${\bar{\sigma }}_{t}-{\bar{\varepsilon }}_{t}$ were developed for the analyzed composites. The true stress (12) and true strain (13) were calculated using the following formulae:

(12) $${\bar{\sigma }}_{t}={\bar{\sigma }}_{x}\left(1+{\bar{\varepsilon }}_{x}\right),$$

(13) $${\bar{\varepsilon }}_{t}=\ln\left(1+{\bar{\varepsilon }}_{x}\right).$$

The effective yield stresses ${\bar{\sigma }}_{ys0.2}$ are determined using the offset yield method. In this approach, a line is drawn on the stress–strain curve parallel (at a 0.2% offset from the origin) to the section of the curve that describes the linear deformation of the material. The yield point is the intersection of this line with the stress–strain curve.

As previously mentioned, the matrix material (in quasi-static analysis) uses an elastic–plastic material with hardening that follows the Hollomon model [56]. Thus, the hardening equation for the composite is expressed as follows:

(14) $${\bar{\sigma }}_{t}={\bar{K}}^{H}{\bar{\varepsilon }}_{tp}^{{\bar{n}}^{H}},$$

where the true plastic strain was calculated using the following formula:

(15) $${\bar{\varepsilon }}_{tp}={\bar{\varepsilon }}_{t}-\frac{{\bar{\sigma }}_{t}}{\bar{E}}.$$

Logarithmizing both sides of Equation (14) yields the following formula:

(16) $$\log\left({\bar{\sigma }}_{t}\right)={\bar{n}}^{H}\log\left({\bar{\varepsilon }}_{tp}\right)+\log\left({\bar{K}}^{H}\right).$$

**Figure 6:**
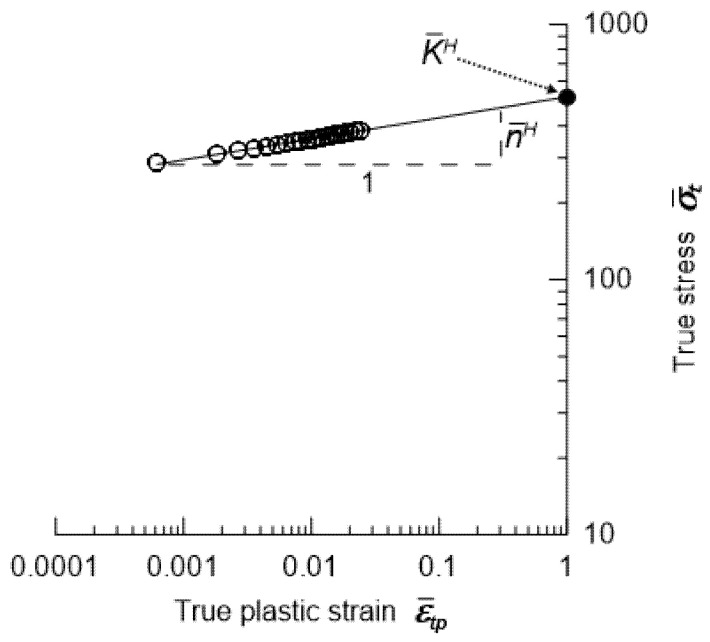
Way of determining two parameters ${\bar{K}}^{H}$ and ${\bar{n}}^{H}$ in Hollomon equation.

The formula above is shown graphically in Figure 6.

The points obtained from Equation (16) can be approximated using a straight line (Figure 6). In practice, the coefficient of determination (R^2^) for these linear fits varies between 0.98 and 0.99, depending on the sample, which confirms the high accuracy of the approximation. The effective strain-hardening exponent ${\bar{n}}^{H}$ is the gradient of this straight line. The effective strength coefficient ${\bar{K}}^{H}$ corresponds to the true stress calculated using Equation (14) (for ${\bar{\varepsilon }}_{tp}$ = 1 and the determined gradient ${\bar{n}}^{H}$).

The last parameter determined was the ultimate tensile strength. Using the chosen material hardening model, this parameter cannot be determined from the graph ${\bar{\sigma }}_{t}-{\bar{\varepsilon }}_{t}$. Therefore, the von Mises criterion was used to determine the effective ultimate tensile strength. In each step of the quasi-static analysis, assuming that *V*_matrix_ and *dV*_matrix_ represent the total and elemental volume of the matrix material, respectively, the Mises-averaged tensile strength was calculated (17):

(17) $${\bar{\sigma }}_{v}=\frac{1}{V_{\mathrm{matrix}}}\int_{V_{\mathrm{matrix}}}\sigma_{v}\;dV_{\mathrm{matrix}},$$

where *σ*_v_ is the local Mises stress [57] in the matrix material.

The safety factor (18) is calculated as follows:

(18) $$FS=\frac{\sigma_{u}}{{\bar{\sigma }}_{v}},$$

where *σ_u_* is the ultimate tensile strength of the matrix.

The ultimate tensile strength was considered to be the averaged stress ${\bar{\sigma }}_{x}$ occurring in the RVE, determined at the step where the safety factor reached 1:

(19) $${\bar{\sigma }}_{u}={\bar{\sigma }}_{x}\left(FS=1\right).$$

Owing to the random distribution and size variation of the reinforcement particles, at least five simulations were performed for each composite configuration, and $\bar{E},\bar{\nu },\bar{\rho },{\bar{\sigma }}_{ys0.2},{\bar{\sigma }}_{u},{\bar{K}}^{H},{\bar{n}}^{H}$ were calculated individually. The average value obtained from the tests was considered the effective value of the determined parameters.

### 2.2. Validation of the Developed Method

To validate the developed method, the effective mechanical properties of the selected composites were determined, and the results were compared with experimental data. These results were compared with the analytical solutions in the absence of experimental data. The analytical solutions include the upper (20) and lower (21) limits of the sought parameter, determined using the rule of mixtures (ROM):

(20) $${\bar{X}}_{u}=\sum_{i=1}^{n}\left(x_{ri}V_{fi}\right)+\left(1-\sum_{i=1}^{n}V_{fi}\right)x_{m}$$

(21) $${\bar{X}}_{l}={\left(\sum_{i=1}^{n}\left(\frac{V_{fi}}{x_{ri}}\right)+\frac{1-\sum_{i=1}^{n}V_{fi}}{x_{m}}\right)}^{-1},$$

where ${\bar{X}}_{u}$ is the effective value of the parameter to be determined, $x_{ri}$ and $x_{m}$ are the values of the parameter for the *ith* reinforcement material and matrix, respectively, $V_{fi}$ is the volume fraction of the *ith* reinforcement material, and *n* is the number of reinforcement fractions.

**Figure 7:**
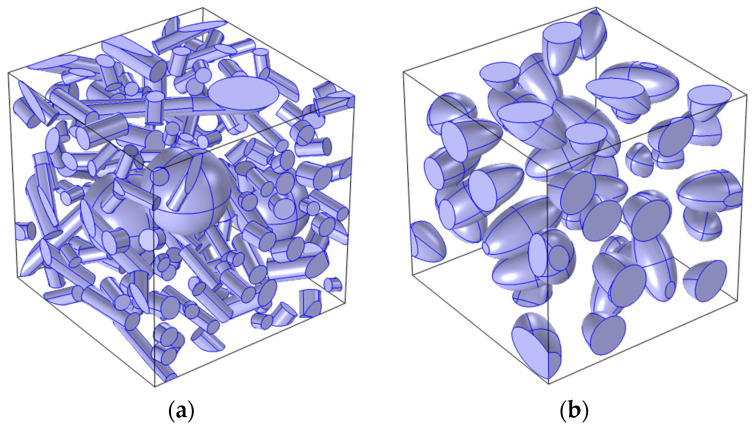
Modeled composites: (**a**) Composite A [53]: Al6061/Al_2_O_3p_ (10%)/Al_2_O_3w_ (10%), (**b**) B [54]: Al6061/Al_2_O_3p_ (20%).

Figure 7 shows the spatial models of the analyzed composites. Two types of composites with the same matrix material (Al6061T6) and different reinforcement variants were selected for the analysis: a hybrid composite (Figure 7a) (reinforced with both short fibers (Al_2_O_3w_) and particles (Al_2_O_3p_), and a composite reinforced with Al_2_O_3p_ particles (Figure 7b).

The experimental results for these composites have been described in detail in previous papers [67,68]. The geometric and mechanical properties of the individual material fractions are listed in Table 1.

The authors of [67,68] did not provide the values of the strain hardening exponent (*n^H^*) and strength coefficient (*K^H^*) for the matrix material. Therefore, in the analyses, these coefficients were adopted based on [69]: *n^H^* = 0.06, *K^H^* = 413.7 MPa.

The selected effective mechanical properties of Composite A were determined using a spatial model (Figure 7a). The results obtained were compared with literature data and analytical solutions (Table 2).

Analyzing the data presented in Table 2, it can be seen that the determined parameters are in good agreement with the literature data.

**Figure 8:**
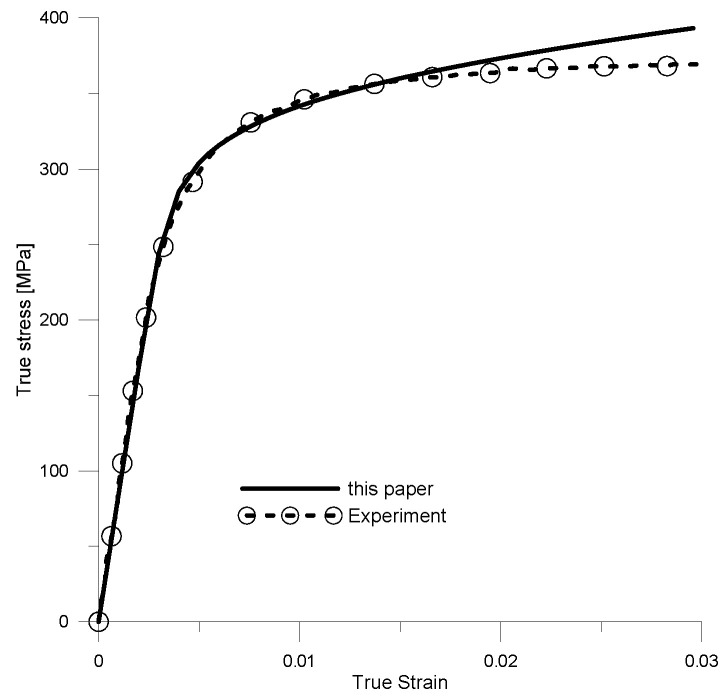
Comparison of stress–strain curves obtained from developed method and experimental studies.

Similar analyses were performed for Composite B. Using the developed spatial model (Figure 7b), the effective mechanical properties were determined. The composite was then modeled as a homogeneous material, described by the determined effective material parameters, and a stress–strain curve was developed. The resulting curve ${\bar{\sigma }}_{t}-{\bar{\varepsilon }}_{t}$ was compared with the flow curve obtained from experimental studies [68] as shown in Figure 8.

A comparison of the two curves shows that the solution obtained is in good agreement with the experimental data over a large range (approximately 1.7% ${\bar{\varepsilon }}_{t}$). Therefore, the developed flow curves can be used to determine parameters such as the Young’s modulus or conventional yield stress. The selected averaged elastic–plastic properties were also determined for Composite B. These properties, together with the experimental data, are listed in Table 3. As with Composite A, the results obtained were in agreement with the experimental data.

In conclusion, the analyses indicate that the developed method provides a reasonably accurate estimation of the effective mechano-physical properties of composites with different reinforcement variants. In most cases, the discrepancy between the estimated parameters and experimentally obtained or analytically derived data did not exceed 3%. However, it is imperative to acknowledge potential sources of error that could influence these results. For instance, certain assumptions made during the modeling process, such as the assumption of perfect bonding between the matrix and reinforcement, as well as the omission of microstructural defects, could contribute to discrepancies between the predicted and experimental values.

Notwithstanding these simplifications, the validation results demonstrated that the proposed method is robust. Overall, the developed approach offers a solid framework for predicting the mechanical behavior of hybrid metal matrix composites.

## 3. Studies on the Influence of the Geometry of the Reinforcement Component on the Effective Properties of Hybrid Composites

This study aimed to determine the influence of the geometrical parameters (shape and size) of the reinforcement particles on the effective properties of hybrid composites. A composite with an aluminium matrix (HAMC) and triple reinforcement was studied. The material properties of the individual components of the composites are listed in Table 4 [70].

**Figure 9:**
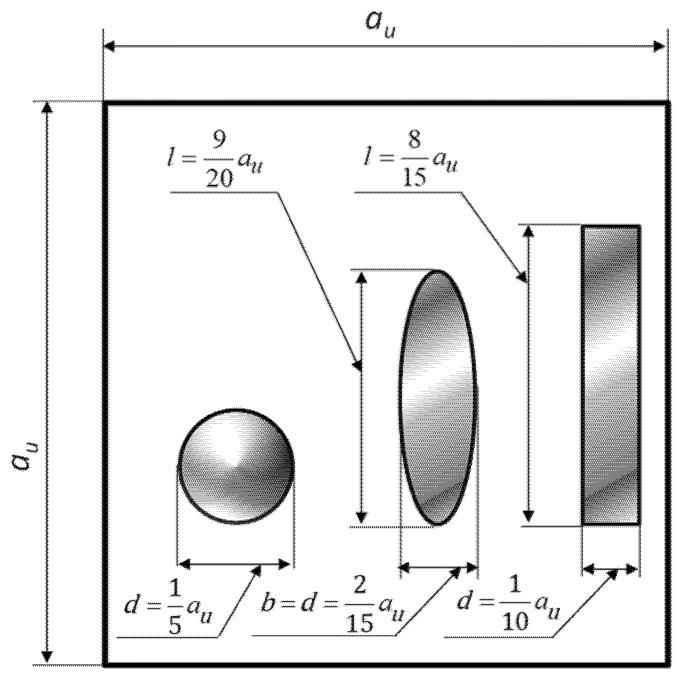
Shape and dimensions of individual reinforcement particles. *d*, *b*, *l*-maximum characteristic dimensions of the reinforcement particles (Figure 1), *a_u_*-characteristic dimension of the RVE (Figure 1).

In the simulations, the shapes and sizes of the reinforcement particles and their volume fractions were varied. The material constants of the individual HAMCs components remain unchanged. The dimensions and shapes of the reinforcements are shown in Figure 9.

The test specimens (geometric and material variants of the HAMCs) used in this study are listed in Table 5.

For Specimens 1–6 (Table 5), it was assumed that the particles of all three reinforcement fractions had the same shape (spheres, cylinders, or ellipsoids) and constant dimensions *d_i_ = d*, *b_i_ = b*, *l_i_ = l*, and *i =* 1 *÷ n*, where *n* is the number of reinforcement particles in the composite. In Specimens 7 to 10, reinforcements of different shapes were used, where Reinforcement 1—sphere, Reinforcement 2—ellipsoid, and Reinforcement 3—cylinder were used. In addition, the characteristic dimensions of the reinforcement particles were varied in Samples 9 and 10. These dimensions were randomly selected from an assumed range: 0.5*d* ≤ *d_i_* ≤ *d*, 0.5*b* ≤ *b_i_* ≤ *b*, and 0.5*l* ≤ *l_i_* ≤ *l*). As previously mentioned, five simulations were performed for each sample.

### 3.1. Influence of Reinforcing Particle Shape on the Effective Properties of Hybrid Composites

Specimens 1–8 were used to investigate the effect of the shape of the reinforcement particles on the effective properties of the HAMCs composite. The averaged elastic–plastic parameters calculated for these specimens at a volume fraction of each reinforcement fraction of *V_p_* = 4% are listed in Table 6.

Analyzing the results presented in Table 6, it can be concluded that the basic effective properties of the composite determined using the developed method are in agreement with the values determined using ROM. The Young’s moduli determined were within the range defined by the upper (21) and lower (20) limits of this parameter. The calculated Poisson’s ratios were identical to the theoretically estimated value (20). The maximum error in the averaged density (SEC-4 *) is less than 1%.

From the results obtained (Table 6), it can also be concluded that the shape of the reinforcement particles influences the effective elastic–plastic parameters of the composite. The highest values of the estimated parameters were obtained when cylindrical reinforcement particles (CCC-4 *) were used for all three reinforcement fractions. Cylindrical particles offer a larger contact area with the composite matrix than spherical or ellipsoidal particles. This increase in the contact area led to a more efficient load transfer and increased the strength of the composite. In contrast, it was smallest for the geometric variant in which the reinforcement particles were of various shapes (SEC-4 *). This result can be attributed to the greater inhomogeneity in the particle distribution, which leads to weakening of the composite structure. Variations in particle shape can cause local stress concentrations, which negatively affects the strength of the material. It is worth noting that this dependence of the effective parameters on the shape of the reinforcement particles is consistent with the experimental results presented in work [23] (the authors studied a hybrid composite with two fractions of reinforcement, with spherical and cylindrical particles, or their combination).

**Figure 10:**
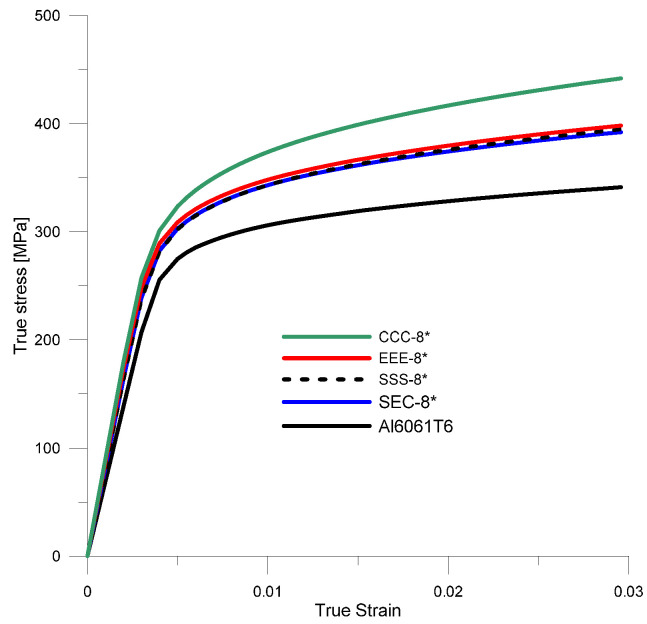
Stress–strain curves for the matrix material (Al6061T6) and the HAMCs composite with a volume fraction of reinforcement *V_p_* = 8% and different shapes of reinforcement particles.

The effective parameters were also determined for composites with a reinforcement volume fraction of *V_p_* = 8% (Figure 10). The effect of the reinforcement shape on the estimated parameters was identical to that of specimens with *V_p_* = 4%.

**Figure 11:**
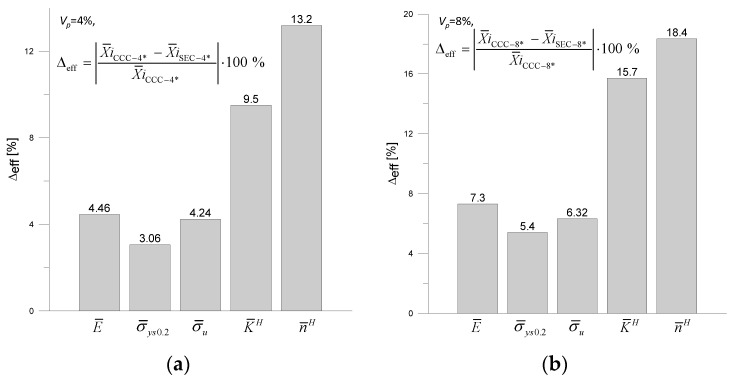
Percentage differences in the effective parameters estimated for the two specimens: (**a**) CCC-4 * i SEC-4 * (*V_p_* = 4%), (**b**) CCC-8 * i SEC-8 * (*V_p_* = 8%).

Figure 11 shows the percentage differences in the averaged parameters $\bar{X}i\left(\bar{E},{\bar{\sigma }}_{ys0.2},{\bar{\sigma }}_{u},{\bar{K}}^{H},{\bar{n}}^{H}\right)$ determined for the specimens with cylindrical (CCC-4 *, CCC-8 *) and varied-shaped (SEC-4 *, SEC-4 *) reinforcements.

By analyzing the data shown in Figure 10, it can be observed that the shape of the reinforcement has the greatest effect on the material hardening parameters ${\bar{K}}^{H}$, ${\bar{n}}^{H}$, and Young’s modulus.

### 3.2. Influence of Reinforcement Particle Size on the Effective Properties of Hybrid Composites

**Figure 12:**
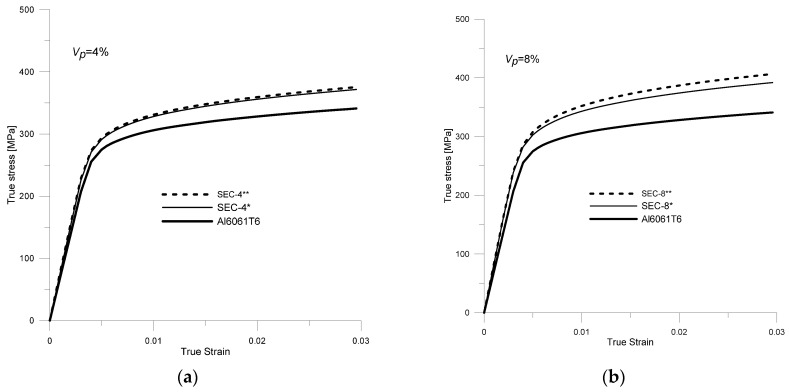
Stress–strain curves for the matrix material (Al6061T6) and the HAMC composite with a volume proportion of reinforcement *V* = 8% and different shapes and sizes of reinforcement particles: (**a**) *V_p_* = 4%, (**b**) *V_p_* = 8%.

To estimate the effect of the reinforcement particle size on the effective properties of the HAMC composite, Samples 7–10 were analyzed (Table 5). Figure 12 shows the stress–strain curves (for specimens in which the characteristic dimensions of the reinforcement particles (*d*, *b*, *l*) were constant (SEC-4 *, SEC-8 *) and for specimens in which the particle dimensions were variable (0.5*d* ≤ *d_i_* ≤ *d*, 0.5*b* ≤ *b_i_* ≤ *b*, 0.5*l* ≤ *l_i_* ≤ *l*) (SEC-4 **, SEC-8 **).

By analyzing these curves (Figure 12), it can be seen that the effective elastic–plastic parameters of the composite are higher when reinforcement particles with smaller characteristic dimensions are used (this relationship is also confirmed by experimental studies on single-reinforcement composites [68]).

**Figure 13:**
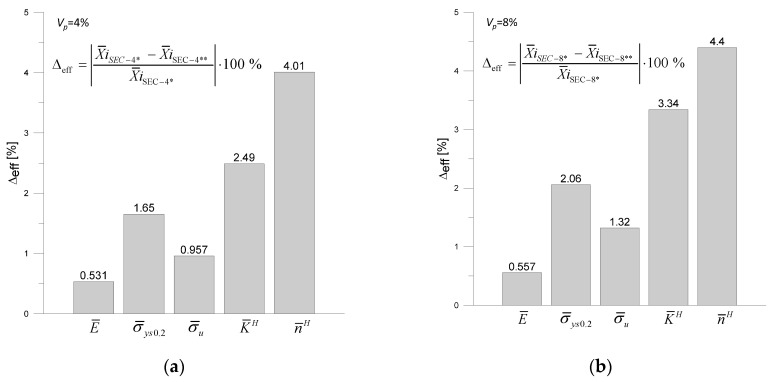
Percentage difference of effective parameters estimated for the two samples: (**a**) SEC-4 * and SEC-4 ** (*V_p_* = 4%), (**b**) SEC-8 * and SEC-8 ** (*V_p_* = 8%).

The increased effective elastic–plastic properties of the composite when smaller reinforcement particles were used were due to several factors. The reduced particle size results in a larger contact area with the matrix per unit volume, which improves stress transfer between the reinforcement and matrix. This, in turn, increases the effectiveness of the composite reinforcement process. In addition, smaller reinforcement particles result in a smaller spacing between the reinforcement particles, which provides a more uniform stress distribution throughout the material. This makes the composite less susceptible to local stress concentrations, which increases its yield and tensile strength. Figure 13 shows the differences in the individual material parameters of the HAMCs composites.

From the results shown in Figure 13, it can be concluded that the shape of the reinforcement has the greatest effect on the material hardening parameters, ${\bar{K}}^{H}$, ${\bar{n}}^{H}$, and yield strength. However, it had the least effect on the Young’s modulus. Moreover, the effect of the size of the reinforcement particles on the studied parameters was much smaller than that of their shape.

### 3.3. Discussion

In summary, the simulation results reveal that the geometry, size, and volume fraction of the reinforcements have a pronounced effect on the effective mechanical properties of the hybrid composite. The reinforcement shape is identified as the most critical parameter. Composites incorporating cylindrical reinforcements consistently exhibit higher effective Young’s modulus, yield strength, and tensile strength compared to those with spherical or ellipsoidal inclusions. This superior performance is attributed to the larger interfacial contact area provided by the cylindrical shape, which promotes more efficient load transfer between the reinforcement and the matrix.

Furthermore, the size of the reinforcements plays an important role in the overall behavior of the composite. Smaller reinforcement particles enhance both the elastic and plastic performance of the composite due to increased surface area and reduced spacing between particles, which results in a more uniform distribution of stress throughout the matrix. In addition, the reinforcement volume fraction exerts a straightforward influence on the mechanical properties: as the volume fraction increases, there is a notable improvement in both stiffness and strength, reflecting the higher contribution of the high-modulus, high-strength reinforcement phase.

These insights suggest that optimizing the reinforcement geometry, size, and volume fraction is crucial for tailoring the mechanical performance of hybrid composites. The clear trend observed—that cylindrical reinforcements offer the most effective enhancement of mechanical properties, followed by the beneficial effect of reduced particle size and increased reinforcement content—provides valuable guidance for the design and development of advanced composite materials.

In the present work, fracture toughness is not simulated. To complement this, we tentatively plan to introduce a fracture with cohesion element layer (CZM) in the RVE to determine J-integral and KIC for different particle shapes and volume fractions.

### 3.4. Method Limitations

In our RVE calculations, a random distribution of particles (spherical, cylindrical, ellipsoidal) with a minimum canter-to-center distance of 1.1 d_max_ is assumed. While this is a standard approximation, it does not account for the actual microstructural features of the specific material under study. The absence of direct comparison with SEM images is a limitation, since the precise distribution of particle sizes and shapes in a real sample may differ from our random model.

In the present model, perfect adhesion between the metal matrix and ceramic inclusions is assumed. In reality, the interface may exhibit finite strength and damaged regions (e.g., deboning or micro-voids), especially under high local stresses or thermal mismatch. Because we do not yet include any cohesive-zone or interfacial-damage formulation, our predictions may overestimate stiffness, yield strength, and ultimate strength. A future extension will introduce a cohesive-zone model at the matrix–inclusion interface, allowing us to define a traction–separation law and critical energy release rate. This will enable simulation of deboning and interfacial crack initiation, yielding a more realistic stress–strain response.

Moreover, when the inclusion volume fraction exceeds the percolation limit, adjacent particles may touch or share very thin matrix ligaments. In such cases, the stress–strain state near those interfaces differs markedly from a fully matrix-separated configuration, and microcracks can nucleate directly between contacting reinforcements. Our present RVE, which enforces a minimum center-to-center distance (1.1 d_max_), cannot capture these particle-to-particle contacts or the associated microdamage. Future work should extend the model by allowing controlled clustering of particles or by incorporating cohesive-zone elements at particle–particle interfaces to simulate micro crack formation.

Modern fabrication techniques for metal matrix hybrids (e.g., casting with solidification, powder metallurgy with sintering, or hot pressing) involve thermal cycles and plastic deformation that generate residual stresses within both the matrix and reinforcement phases. These locked-in stresses shift the initial stress–strain curve and can either raise or lower the apparent yield strength, hardening behavior, and stiffness of the composite under subsequent loading. Because our current RVE assumes a stress-free initial state, it does not capture these process-induced residual stresses. Future work will integrate residual stress fields—derived from manufacturing process simulations or experimental characterization methods—into the RVE to assess their influence on predicted effective properties.

Our current model also assumes that the metallic matrix deforms according to bulk elasto-plastic laws (von Mises yield and Hollomon hardening). However, when the metal ligament thickness between ceramic inclusions falls below approximately 50–100 nm, conventional dislocation-mediated plasticity becomes suppressed due to dislocation starvation and surface-dominated effects. In this nanoscale regime, the yield strength can increase dramatically and hardening behavior changes, so the continuum plasticity framework no longer holds. Therefore, the present RVE predictions are valid only when matrix ligament thicknesses exceed this critical size. Future extensions will consider size-dependent plasticity formulations (for example, strain-gradient plasticity or source-limited models) to capture elastic–plastic behavior in composites with ultrathin matrix regions.

## 4. Summary and Conclusions

### 4.1. Developed Analytical–Numerical Method

This paper presents an analytical–numerical method for determining the effective mechanical properties of hybrid composites. The method enables spatial modeling of composite microstructures with various reinforcement forms (spheres, cylinders, ellipsoids). Reinforcement particle locations and dimensions are randomly generated within defined ranges.

### 4.2. Validation Cases

Two composite types were analyzed for validation:

- A traditional composite with a single reinforcement fraction.
- A hybrid composite with dual reinforcement.

In both cases, the results agreed with experimental data and analytical solutions (e.g., rule of mixtures).

### 4.3. Influence of Particle Geometry

Investigations assessed how reinforcement particle geometry and size affect effective mechanical properties. Findings included:

- Cylindrical particles produced the highest values for Young’s modulus, yield strength, and tensile strength.
- Mixed or varied shapes yielded lower mechanical properties.
- Smaller particles improved hardening and yield strength, though their effect on Young’s modulus was minimal.

### 4.4. Method Limitations

- The random particle configuration requires multiple simulations to ensure statistical reliability, increasing computational effort.
- Modeling was restricted to primitive rotating solids (spheres, cylinders, ellipsoids), limiting accuracy for composites with more complex particle geometries.
- Complex microstructural interactions between particles were not considered, which may influence results in advanced cases.

### 4.5. Practical Relevance

The proposed method is valuable for materials engineers and researchers in aerospace, automotive, and related industries. It provides tools to model and predict composite properties accurately, facilitating the design of materials with optimized mechanical performance.

### 4.6. Future Work

To further improve mechanical property predictions for HMMCs, future research should consider integrating a U-Net architecture. This approach would learn stress–strain partitions from crystal plasticity simulations and then predict mechanical parameters at minimal computational cost [71].

## Figures and Tables

**Table 1:** Geometry and mechano-physical properties of materials used for the matrix and reinforcement [53,54].

Material		Young Modulus *E* [GPa]	Poisson Ratio ν	Density *ρ* [kg/m^3^]	Yield Stress *σ_ys_*_0.2_ [MPa]	Ultimate Tensile Strength *σ_u_* [MPa]	Diameter *d* [μm]	Diameter b [μm]	Length *l* [μm]
Composite A [67]	Al6061	68.4	0.33	2700	208.3	299.6	-	-	-
	Al_2_O_3p_	380	0.21	3950	-	-	45	-	-
	Al_2_O_3w_	310	0.21	3300	-	-	1.5–6.6	-	3–110
Composite B [68]	Al6061	69	0.33	2700	276	310	-	-	-
	Al_2_O_3p_	297	0.21	3720	-	-	7.5–10	7.5–10	15–20

**Table 2:** Effective mechanical properties determined for Composite A (Al6061/Al_2_O_3p_ (10%)/Al_2_O_3w_ (10%)).

	Young Modulus $\bar{E}$ [GPa]	Poisson Ratio $\bar{\nu }$	Density $\bar{\rho }$ [kg/m^3^]	Yield Stress ${\bar{\sigma }}_{ys0.2}$ [MPa]	Ultimate Tensile Strength ${\bar{\sigma }}_{u}$ [MPa]
This paper	93.2 ± 0.3	0.31 ± 0.01	2865.9 ± 0.8	322.1 ± 1.5	403.8 ± 3
Experiment [53]	94.8 ± 0.6	-	-	318.3 ± 4.5	408 ± 8.9
Upper limit (20)	123.7	0.31	2885	-	-
Lower limit (21)	81.4	-	-	-	-

**Table 3:** Effective mechanical properties determined for Composite B.

	Poisson Ratio $\bar{\nu }$	Ultimate Tensile Strength ${\bar{\sigma }}_{u}$ [MPa]	Strength Coefficient ${\bar{K}}^{H}$ [MPa]	Strain Hardening Exponent ${\bar{n}}^{H}$
experiment [68]	0.31 ± 0.02	370 ± 5	-	-
this paper	0.31 ± 0.01	360 ± 2	520.63	0.0825

**Table 4:** Mechano-physical properties of the materials used for the matrix and reinforcement of the HAMCs composite.

Material	Young Modulus *E* [GPa]	Poisson Ratio ν	Density *ρ* [kg/m^3^]	Yield Stress *σ_ys_*_0.2_ [MPa]	Ultimate Tensile Strength *σ_u_* [MPa]	Strength Coefficient *K^H^* [MPa].	Strain Hardening Exponent *n^H^*
matrix (Al6061T6)	69	0.33	2700	276	310	413	0.06
reinforcement 1 (BN)	100	0.21	2250	-	-	-	-
reinforcement 2 (Al_2_O_3_)	297	0.21	3720		-	-	-
reinforcement 3 (SiC)	480	0.31	4900	-	-	-	-

**Table 5:** Test specimens.

		Reinforcement 1		Reinforcement 2		Reinforcement 3	
No	Sample	Shape	Volume Fraction *V_p_* [%]	Shape	Volume Fraction *V_p_* [%]	Shape	Volume Fraction *V_p_* [%]
1	SSS-4 *	S	4	S	4	S	4
2	SSS-8 *	S	8	S	8	S	8
3	CCC-4 *	C	4	C	4	C	4
4	CCC-8 *	C	8	C	8	C	8
5	EEE-4 *	E	4	E	4	E	4
6	EEE-8 *	E	8	E	8	E	8
7	SEC-4 *	S	4	E	4	C	4
8	SEC-8 *	S	8	E	8	C	8
9	SEC-4 **	S	4	E	4	C	4
10	SEC-8 **	S	8	E	8	C	8

*−*d_i_* = *d*, *b_i_* = *b*, *l_i_* = *l*; **−0.5*d* ≤ *d_i_* ≤ *d*, 0.5*b* ≤ *b_i_* ≤ *b*, 0.5*l* ≤ *l_i_* ≤ *l*; shape: S-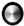, C-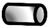, E-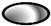.

**Table 6:** The effective mechanical properties of the HAMCs composite were calculated for *V_p_* = 4%.

Material	Young Modulus $\bar{E}$ [GPa]	Poisson Ratio $\bar{\nu }$	Density $\bar{\rho }$ [kg·m^−3^]	Yield Stress ${\bar{\sigma }}_{ys0.2}$ [MPa]	Ultimate Tensile Strength ${\bar{\sigma }}_{u}$ [MPa]	Strength Coefficient ${\bar{K}}^{H}$ [MPa]	Strain Hardening Exponent ${\bar{n}}^{H}$
SEC-4 *	76.53 ± 0.4	0.32 ± 7·10^−4^	2787.73 ± 3.9	302.36 ± 0.6	332.54 ± 0.4	494.95 ± 3.2	0.079 ± 1·10^−3^
SSS-4 *	77.73 ± 0.6	0.32 ± 5·10^−4^	2791.30 ± 7.6	303.25 ± 0.8	334.85 ± 0.9	501.61 ± 1.5	0.080 ± 1·10^−4^
EEE-4 *	78.63 ± 0.76	0.32 ± 5·10^−4^	2796.77 ± 8.2	305.03 ± 0.9	335.89 ± 1.1	505.94 ± 5.2	0.081 ± 1·10^−3^
CCC-4 *	80.10 ± 0.15	0.32 ± 1·10^−3^	2817.03 ± 4.3	311.90 ± 0.6	347.25 ± 1.8	546.89 ± 6.2	0.091 ± 1·10^−3^

${\bar{\rho }}_{t}$ = 2810.8, ${\bar{\nu }}_{t}$ = 0.32, ${\bar{E}}_{L}$ = 74.79, ${\bar{E}}_{U}$ = 95.8; ${\bar{\rho }}_{t},{\bar{\nu }}_{t}$—theoretical density and Poisson ratio calculated based on ROM (20); ${\bar{E}}_{L},{\bar{E}}_{U}$—lower (20) and upper (21) Young’s modulus limit calculated based on ROM.

## Data Availability

The original contributions presented in the study are included in the article, further inquiries can be directed to the corresponding author.
